# Supplementary material for: Inhibition of glucose metabolism selectively targets autoreactive follicular helper T cells
Source: Nat Commun. 2018 Oct 22;9:4369. doi: 10.1038/s41467-018-06686-0 (PMC6197193; doi:10.1038/s41467-018-06686-0)
Supplement: Supplementary file 1 — Supplementary Information [file 41467_2018_6686_MOESM1_ESM.pdf]

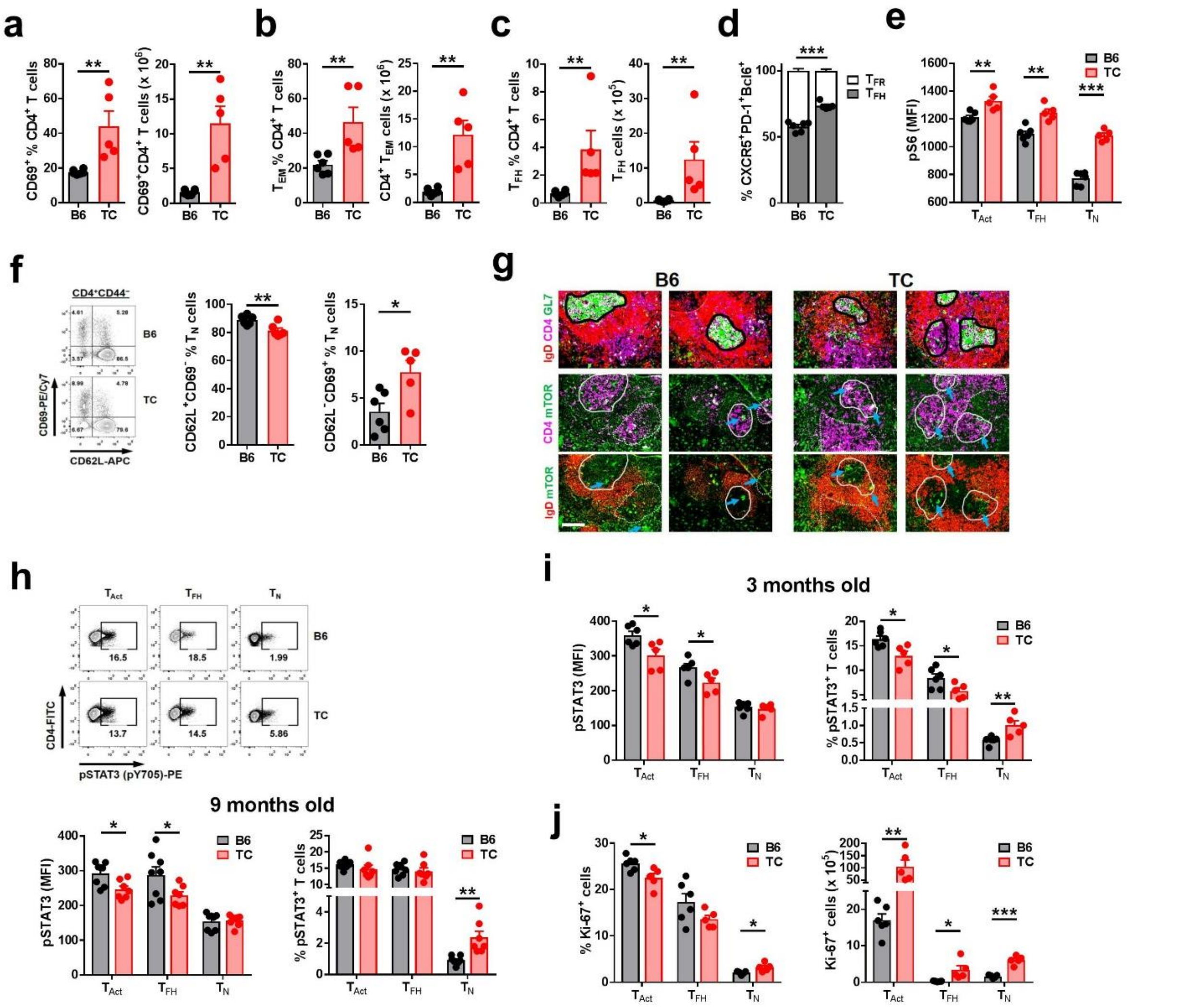

Supplementary Fig. 1 Increased activation and mTORC1 activity in splenic CD4^{+} T cells from pre-autoimmune lupus mice. Frequency and absolute number of CD69^{+} cells (**a**), T_{EM} cells (**b**) and T_{FH} cells (**c**) in CD4^{+} T cells, and relative distribution of T_{FH} and T_{FR} cells shown as stacked columns. For clarity, the individual values for T_{FR} samples are not shown. (**d**). **e** Phospho-S6 MFI in T_N, T_{Act} and T_{FH} cells. **f** Frequency of CD62L^{+}CD69^{-} and CD62L^{-}CD69^{+} in T_N cells from aged mice. **g** Representative immunofluorescence staining in spleens of B6 and TC mice. The top row shows GL7^{+} (green) positive GCs containing CD4^{+} T cells (purple) but excluding IgD^{+} (red) B cells, outlined with a black line. The same GCs are shown below with mTOR (green) staining relative to CD4^{+} T cells (middle) and to non-GC B cells (bottom). GCs are outlined with a plain line and the T cell zone with a dashed line. Arrows point to representative mTOR staining. All images are shown with a 20x magnification (scale bar: 100uM). Representative pSTAT3 staining in B6 and TC $T_{Act,}$ T_{FH} and T_N cells, and pSTAT3 MFI (middle) and frequency (right) in aged (**h**) and young (**i**) mice. **j** Frequency and number of Ki-67^{+} proliferating $T_{Act,}$ T_{FH} and T_N cells, rom 3 month-old B6 and TC mice. Mean + s. e. m. of N = 3 – 8 mice per group compared with t tests. *$P < 0.05$, **$P < 0.01$, and ***$P < 0.001$.

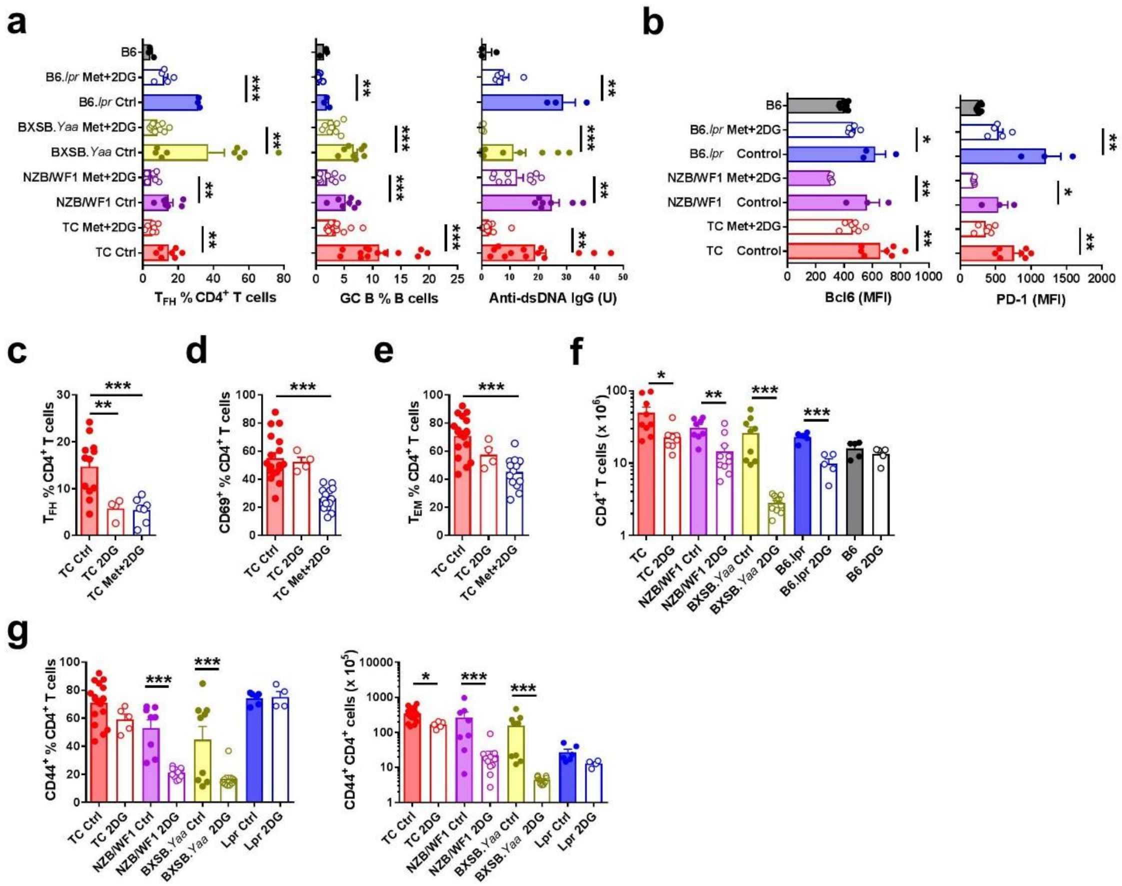

Supplementary Fig. 2 The addition of metformin does not enhance the effect of glycolysis inhibition on T_{FH} cells from lupus-prone mice. Anti-dsDNA IgG positive lupus-prone mice from the indicated strains were treated with Metformin + 2DG for 8 weeks and compared to untreated age-matched controls. Contemporaneous untreated B6 mice are shown as reference. **a** Frequency of T_{FH} cells (left, $CD4^+CXCR5^+PD1^+Bcl6^+Foxp3^-$) in splenic $CD4^+$ T cells and GC B cells (middle, $B220^+GL7^+Fas^+$) in B cells, as well as serum anti-dsDNA IgG (right). **b** Bcl6 and PD-1 MFI on total $CD4^+$ T cells. Frequency of T_{FH} cells (**c**), $CD69^+$ cells (**d**), and T_{EM} cells (**e**) in $CD4^+$ T cells from 6 month-old TC mice treated for 8 weeks with 2DG, the combination of Metformin + 2DG, or untreated controls. **f** Number of total $CD4^+$ T cells. **g** Frequency and number of $CD44^+$ $CD4^+$ T cells for each of the lupus strain treated or not with 2DG. Mean + s. e. m. of N = 4 – 18 mice per group compared with t tests. $^*P < 0.05$, $^{**}P < 0.01$, and $^{***}P < 0.001$. Statistical comparisons are within strains between treated and control mice.

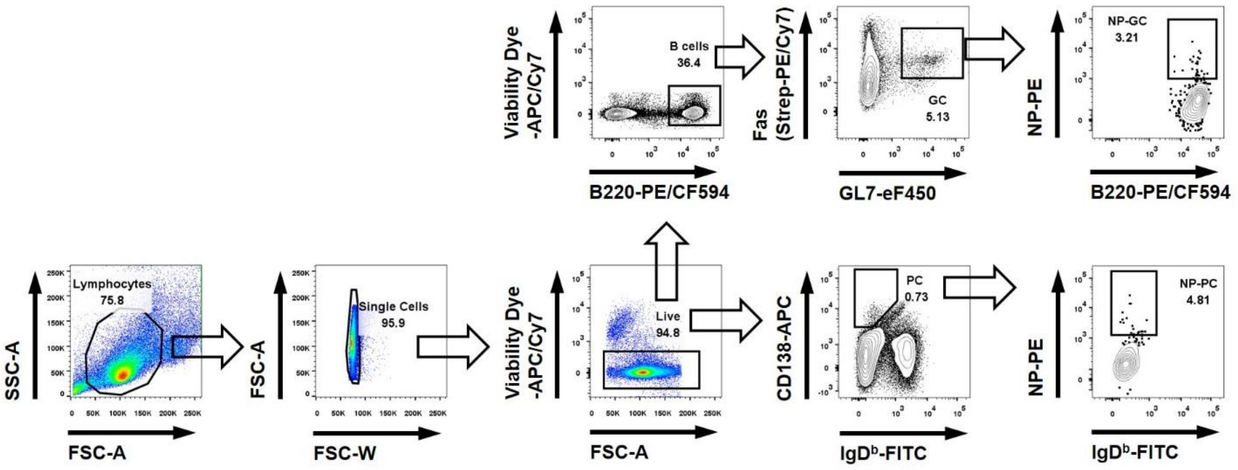

Supplementary Fig. 3 Representative gating strategy for splenic GC B cells and plasma cells used in Figure 3.

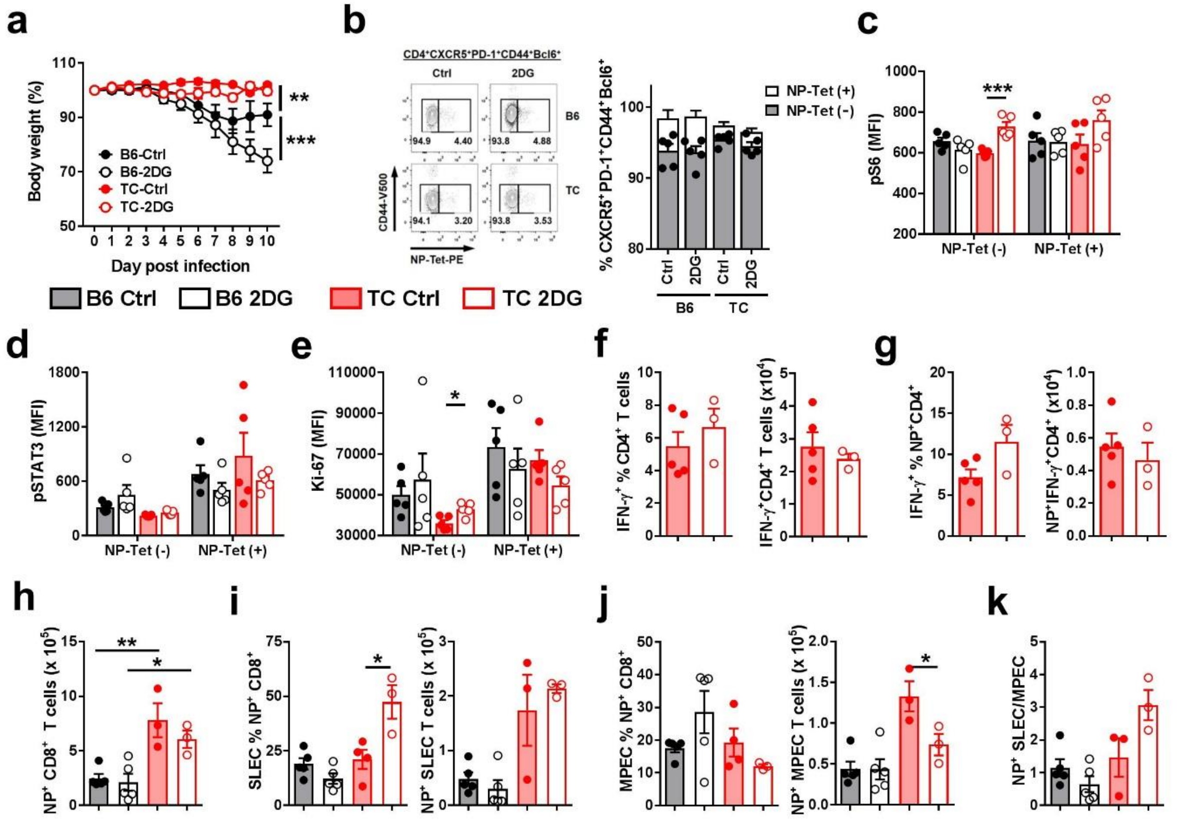

Supplementary Fig. 4 Effect of glycolysis inhibition on T cell responses to influenza virus**.** B6 and TC mice were infected with PR8 influenza virus. 2DG treatment was initiated two weeks before infection and maintained until termination at d 10 post infection. **a** Body weight loss. The graph shows means + s. e. m. of N = 5 per group. Comparisons were made between untreated B6 and TC mice, and between treated and control B6 mice by 2-way ANOVA. **b** Representative FACS plots (left) and frequencies shown as stacked columns (right) of NP-specific and NP-Tet^{neg} T_{FH} cells gated as $CD4^+CD44^+CXCR5^+PD\text{-}1^+Bcl6^+$. For clarity, the individual values for the NP-Tet$^+$ samples are not shown. MFI of phospho-S6 (**c**), phospho-STAT3 (**d**), and Ki-67 (**e**) were analyzed in influenza virus NP-specific ($CD4^+CD44^+CXCR5^+PD\text{-}1^+Bcl6^+$NP-Tet^{pos}) and non-specific ($CD4^+CD44^+CXCR5^+PD\text{-}1^+Bcl6^+$NP-Tet^{neg}) T_{FH} cells. **f-g** Frequency and number of IFNγ^+CD4^+ T cells (**f**) and IFNγ^+NP-Tet^{pos} $CD4^+$ T cells (**g**) in the spleen of 2DG-treated TC mice and controls. **h-k** Analysis of NP-Tet^{pos} $CD8^+$ T cells in the spleen of 2DG-treated B6 and TC mice and controls. Total number of cells (**h**), frequency and number of SLEC NP-Tet^{pos} $CD8^+$ T cells (**i**) and MPEC NP-Tet^{pos} $CD8^+$ T cells (**j**), and NP-Tet^{pos} SLEC/MEPC ratio (**k**). b-k: Mean + s. e. m. of N = 3-5 mice per group compared with t tests. **P* < 0.05, ***P* < 0.01, and ****P* < 0.001.

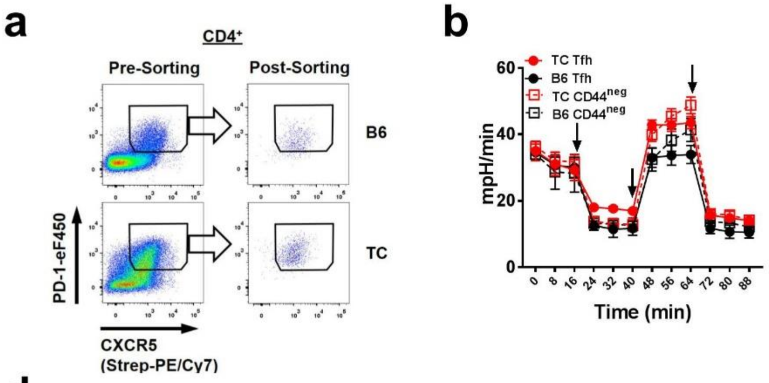

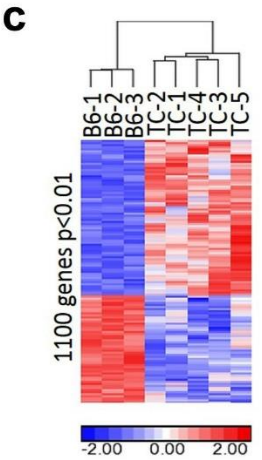

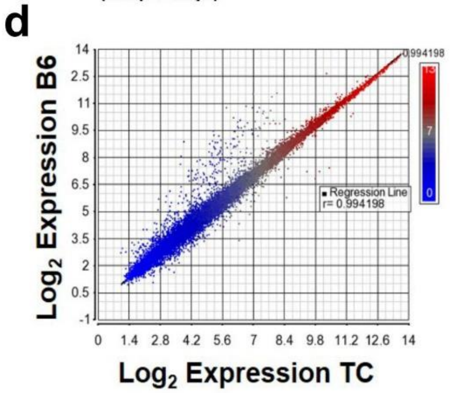

e

Gene Set Enrichment pathways differentially expressed between spontaneous TC and B6 T_{FH} cells.

	Enrichment P-value	Genes Enriched / Entire Pathway
E2F_TARGETS	<0.001	93/192
G2M_CHECKPOINT	<0.001	91/193
OXIDATIVE_PHOSPHORYLATION	0.026	115/189
MTORC1_SIGNALING	0.030	83/190
KRAS_SIGNALING_DN	0.038	106/190
IL6_JAK_STAT3_SIGNALING	0.045	16/85
MYC_TARGETS_V2	0.057	24/58

f

Top 10 differentially expressed genes based on P value

Gene		P value	Fold TC/B6
Tceanc2	transcription elongation factor A (SII) N-terminal and central domain containing 2	2.92E-07	-2.406
Ptpn5	protein tyrosine phosphatase, non-receptor type 5	3.52E-07	-2.439
Iglon5	IgLON family member 5	4.64E-06	1.933
Slc6a16	**solute carrier family 6, member 16**	**7.60E-06**	**1.580**
Slc12a8	**solute carrier family 12 (potassium/chloride transporters), member 8**	**8.40E-06**	**-1.174**
Slc1a5	**solute carrier family 1 (neutral amino acid transporter), member 5**	**8.96E-06**	**-1.840**
Vmn2r29	vomeronasal 2, receptor 29	1.02E-05	-1.589
Igsf23	immunoglobulin superfamily, member 23	1.21E-05	2.455
Syt14	synaptotagmin XIV	1.28E-05	2.392
Htatip2	HIV-1 tat interactive protein 2, homolog	3.00E-05	-3.648

Supplementary Fig. 5 Comparison of metabolism and gene expression between TC and B6 T_{FH} cells. **a** Gating strategy to sort $CD4^+CXCR5^+PD\text{-}1^+$ T_{FH} cells from the spleen of 7-8 month-old TC and B6 mice. **b** OCR during a mitochondrial stress test conducted on T_{FH} ($CD4^+CD44^+PD\text{-}1^{hi}PSGL\text{-}1^{lo}$) and $CD44^-CD4^+$ T cells, with the arrows indicating from left to right the addition of oligomycin, FCCP, and actimycin A plus rotenone. **c-d** Microarray results of the comparison between spontaneous T_{FH} cells sorted from B6 (left) and TC (right) mice shown as a heat map (**c**) and scatter plot (**d**). Heatmap coloring is mean centered (white), standard deviation normalized to 1 with red indicating above average expression and blue for below average expression (c). Dotplot coloring is based on signal intensity Robust Microarray Average (RMV) normalized values, blue is lowest and red is highest expression (d). All values from 0 to 14 are shown. **e-f** Gene Set Enrichment pathways differentially expressed between the two strains (**e**) and top 10 differentially expressed genes (**f**) based on the microarray results. Bold face indicate solute transporter genes.

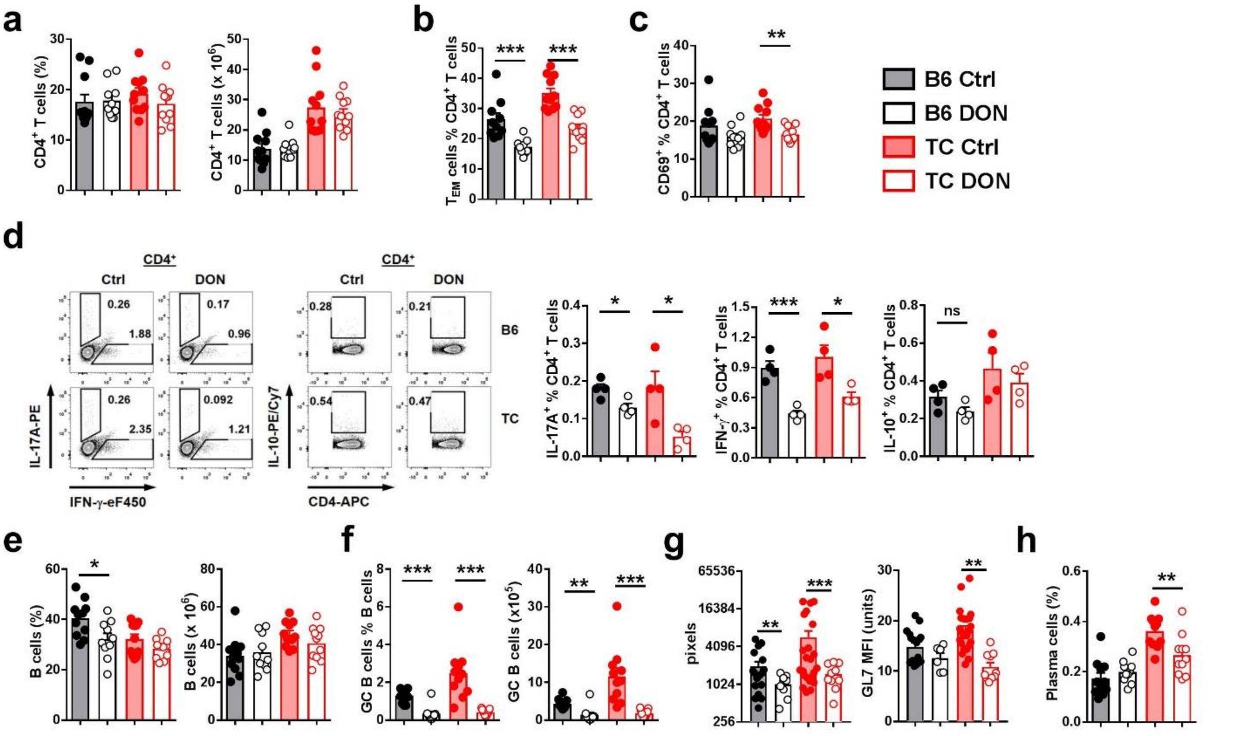

Supplementary Fig. 6 Effect of DON on TD-immunized mice. B6 and TC mice were immunized with NP-KLH in alum and treated with DON, then analyzed at 10 d after immunization as shown in Fig. 7a. **a** Frequency and number of total $CD4^+$ T cells. Frequency of T_{EM} cells (**b**) and $CD69^+CD4^+$ cells (**c**). **d** After stimulating splenocytes with PMA and ionomycin in the presence of GolgiStop, $CD4^+$ T cells were analyzed for the frequency of IL-$17A^+$, IFNγ^+, and IL-10^+ cells. Representative FACS plots of IL-$17A^+$, IFNγ^+, and IL-10^+ cells are shown on the left. **e-f** Frequency and number of total B cells (**e**) and GC B cells (**f**). **g** GC surface area as measured by $GL7^+$ pixels (left) and GL7 intensity in these areas (right), in 3-5 high power fields from 2 mice per group. **h** Absolute number of plasma cells. All cells were analyzed from spleens. Mean + s. e. m. of N = 4-11 mice per group (except g) compared with t tests. **P* < 0.05, ***P* < 0.01, and ****P* < 0.001.

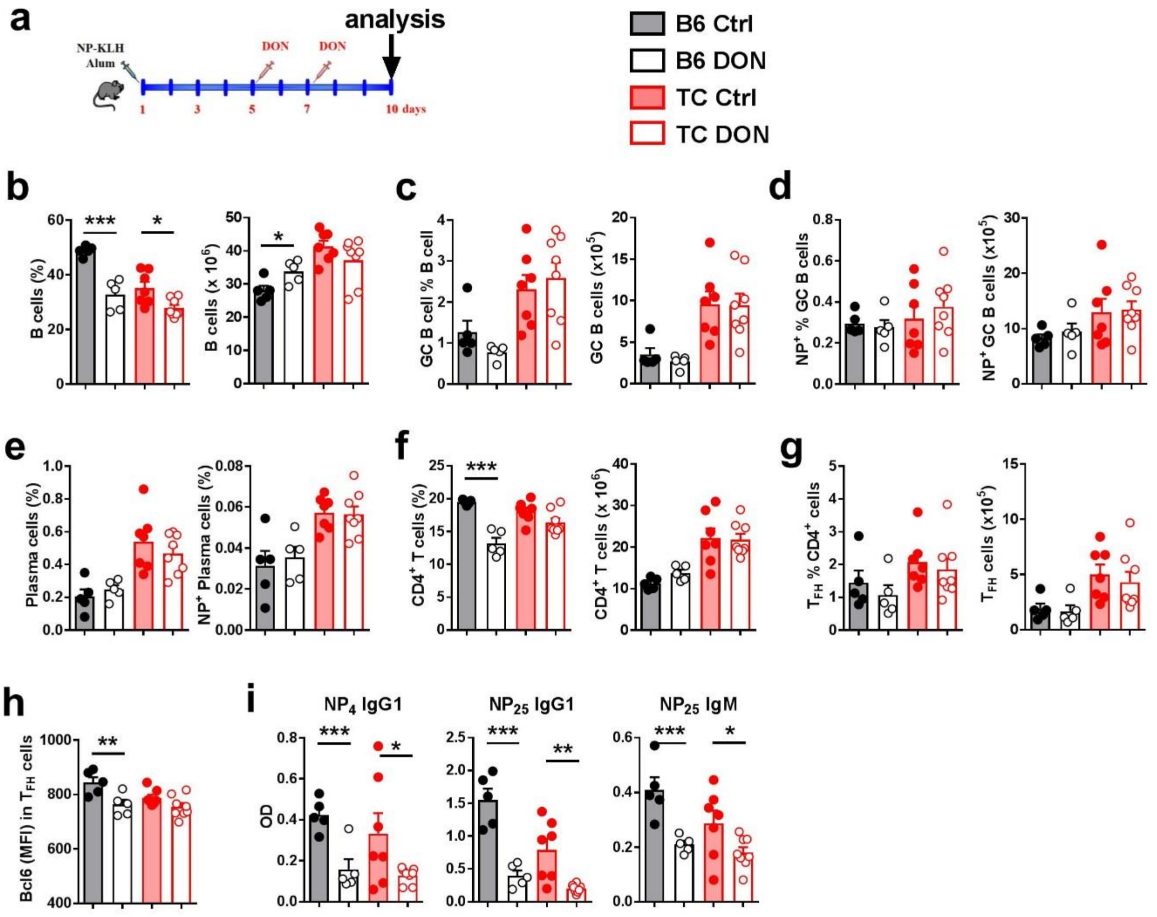

Supplementary Fig. 7 Effect of DON treatment at the time of GC formation on TD response. **a** Experimental design for immunization with NP-KLH in alum and treatment with glutamine antagonist DON in B6 and TC mice. **b-d** Frequency and number of total B cells (**b**), and total (**c**) or NP-specific (**d**) GC B cells. **e** Frequency of total and NP-specific plasma cells. Frequency and number of total CD4$^+$ T cells (**f**) and T_{FH} cells (**g**). **h** MFI of Bcl6 in T_{FH} cells. Serum levels of high affinity anti-NP_4, low affinity anti-NP_{25} IgG1, and IgM NP-specific antibodies (**i**), and anti-dsDNA IgG (j). All cells were analyzed from spleens. Mean + s. e. m. of N = 5-8 mice per group compared with t tests. *P < 0.05, **P < 0.01, and ***P < 0.001.

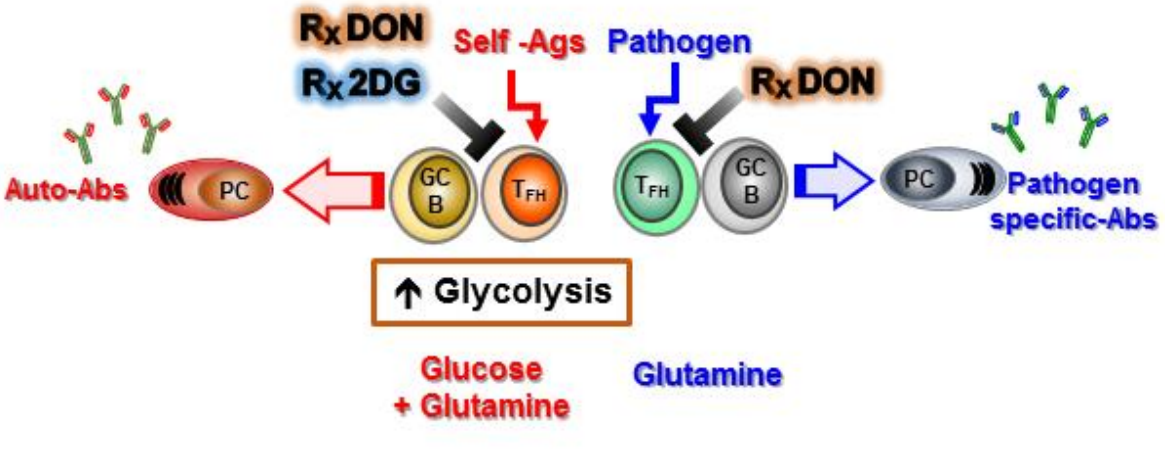

Supplementary Fig. 8 The differentiation of autoreactive T_{FH} and Ag-specific T_{FH} is driven by different metabolic programs. Results presented in this study support the following working model: The production of class-switched antibodies, either in response to TD-antigens or autoantigens requires glutamine and can be blocked with DON. In addition, spontaneous differentiation and expansion of T_{FH} cells in lupus-prone mice depends on glucose metabolism. This process and the subsequent GC B cell expansion and autoantibody production can be blocked with 2DG. On the other hand, exogenous Ag or pathogen-driven T_{FH} differentiation and expansion is glucose-independent, and therefore not affected by 2DG.

Supplementary Table 1. Solute transporter genes in microarray

Gene Symbol	RefSeq	Common Name/Alias	Family/Class	*p*-value	Mean(B6)	Mean(TC)	Fold Change (B6/TC)
Slc1a4	NM_018861	Asct1	Glutamate Transporters	0.000773	5.66315	6.7095	-2.0653
Slc6a16	XM_355900		Sodium and Chloride Symporters	7.60E-06	3.99995	4.65977	-1.57988
Slc37a2	NM_001145960	Ci2	Sugar/Phosphate Exchanger	0.027843	6.63055	7.15405	-1.43744
Slc16a10	NM_001114332	Mct10	Monocarboxylate Transporters	0.016609	5.96864	6.38305	-1.33276
Slc38a6	NM_001037717		Neutral aa Transporters	0.000384	6.01913	6.31227	-1.22531
Slc22a15	NM_001039371		Cation/Anion/Zwitterion Transporters	0.043912	7.52101	7.75334	-1.17473
Slc2a6	NM_001177627	Glut6	Glucose Transporter Class III	0.022024	6.0159	6.24314	-1.17059
Slco2a1	NM_033314	Pgt	Organic Anion Transporters	0.011966	4.42929	4.62264	-1.14342
Slc16a3	NM_001038653	Mct3	Monocarboxylate Transporters	0.037375	6.29594	6.47036	-1.12851
Slc6a13	NM_144512	Gat3	Sodium and Chloride Symporters	0.017071	4.32363	4.49628	-1.12713
Slco3a1	NM_001038643	Anr1	Organic Anion Transporters	0.024044	7.1862	7.35027	-1.12044
Slc22a17	NM_021551	Boit	Cation/Anion/Zwitterion Transporters	0.043028	6.04775	6.20162	-1.11255
Slc26a9	NM_177243		Anion Exchanger	0.034369	4.10769	4.25696	-1.10901
Slc25a18	NM_001081048		Mitochondrial	0.033406	4.82682	4.94371	-1.08439
Slc5a10	NM_001033227	Sglt5	Sodium Glucose Cotransporters	0.048219	4.4636	4.57481	-1.08013
Slc2a13	NM_001033633	Glut13, Hmit	Glucose Transporter Class III	0.043971	4.08132	4.19247	-1.08008
Slc13a4	NM_172892	Sut1	Sodium Sulfate/Carboxylate Cotransporters	0.041359	4.21033	4.2763	-1.04679
Slc5a6	NM_001177621		Sodium Glucose Cotransporters	0.038632	5.84172	5.72638	1.08323
Slc35b1	NM_016752	Ugtrel1	Nucleoside Sugar Transporters	0.028039	7.00047	6.84446	1.1142
Slc52a3	NM_001164819	Rft2	Riboflavin Transporter	0.0128	7.19199	7.02814	1.12027
Slc7a5	NM_011404	Lat1 (CD98)	Fatty Acid Transporter	0.010204	7.22403	7.04819	1.12962
Slc12a8	NM_001083902	Ccc9	Cation-Chloride Cotransporter	8.40E-06	5.00232	4.77056	1.17426
Slc20a2	NM_011394	Pit2	Sodium-Phosphate Cotransporters	0.008263	7.09636	6.85588	1.18138
Slc35d1	NM_177732	Ugtrel7	Nucleoside Sugar Transporters	0.016514	5.98879	5.7228	1.20246
Slc28a2	NM_172980	Cnt2	Sodium Nucleoside Transporters	0.019055	8.60788	8.28789	1.24832
Slc14a1	NM_001171010	Utb1	Urea Transporters	0.00656	7.43778	7.07222	1.28838

Gene Symbol	RefSeq	Common Name/Alias	Family/Class	*p*-value	Mean(B6)	Mean(TC)	Fold Change (B6/TC)
Slc43a3	NM_021398	Eeg1	System L aa Transporters	0.003683	7.60423	7.21813	1.30685
Slc7a10	NM_017394	Asc-1	Heterodimeric aa Transporters	0.010705	7.27029	6.56092	1.63509
Slc1a5	NM_009201	Asct2	Glutamate Transporters	8.96E-06	8.50362	7.62381	1.84013
Slc43a1	NM_001081349	Lat3	System L aa Transporters	0.004064	6.47416	5.55887	1.88595
Slc15a2	NM_001145899	Pept2	Proton Oligopeptide Cotransporter	0.029135	6.08086	5.0802	2.00091

Supplementary Table 2. nCounter^{TM} CodeSet Design

HUGO Gene	Accession	Position	Ect
Ahr	NM_013464.4	1328-1427	
Ascl2	NM_008554.2	346-445	
Bcl2	NM_009741.3	1845-1944	
Bcl6	NM_009744.3	186-285	
Calm3	NM_007590.3	1981-2080	
Camk2d	NM_001025439.1	1316-1415	
Ccnb1	NM_172301.3	2137-2236	
Ccr4	NM_009916.2	395-494	
Ccr6	NM_001190333.1	661-760	
Cd109	NM_153098.3	2721-2820	
Cd48	NM_007649.4	371-470	
Cdc25c	NM_009860.2	43-142	
Cdc26	NM_139291.3	313-412	
Cdkn3	NM_028222.1	279-378	
Cpt1c	NM_001252470.1	1135-1234	
Csf1	NM_001113530.1	834-933	
Cx3cr1	NM_009987.3	2697-2796	
Cxcr5	NM_007551.2	1649-1748	
Cyp1b1	NM_009994.1	115-214	
Cyp51	NM_020010.2	2186-2285	
Dhdh	NM_027903.3	571-670	
Dusp6	NM_026268.2	1649-1748	
Egr2	NM_010118.2	1786-1885	
Ezh2	NM_007971.2	426-525	
Fbxo5	NM_025995.2	476-575	
Foxo1	NM_019739.2	2531-2630	

HUGO Gene	Accession	Position	Ect
Foxo3	NM_019740.2	2222-2321	
Foxp3	NM_054039.2	195-294	
Galm	NM_176963.4	175-274	
Gata3	NM_008091.3	1944-2043	
Gusb	NM_010368.1	1736-1835	Housekeeping
Hdac9	NM_024124.2	1521-1620	
Hif1a	NM_010431.2	1295-1394	
Hprt	NM_013556.2	31-130	Housekeeping
Hsd17b7	NM_010476.3	677-776	
Ifitm1	NM_001112715.1	413-512	
Ifitm2	NM_030694.1	10-109	
Ifnar1	NM_010508.1	1196-1295	
Ifng	NM_008337.1	96-195	
Igf2r	NM_010515.1	2586-2685	
Il13	NM_008355.2	426-525	
Il13ra1	NM_133990.4	846-945	
Il17a	NM_010552.3	206-305	
Il21	NM_021782.2	1763-1862	
Il22	NM_016971.1	478-577	
Il23r	NM_144548.1	691-790	
Il4	NM_021283.1	346-445	
Il6	NM_031168.1	41-140	
Il9r	NM_008374.2	2818-2917	
Irf4	NM_013674.1	1879-1978	
Itgae	NM_008399.1	776-875	
Kif11	NM_010615.1	4176-4275	

HUGO Gene	Accession	Position	Ect
Lbr	NM_133815.1	1196-1295	
Ldhd	NM_027570.3	421-520	
Lrig1	NM_008377.2	4356-4455	
Msmo1	NM_025436.2	201-300	
Mtus1	NM_001005863.2	4231-4330	
Nr4a1	NM_010444.1	1316-1415	
Nr4a2	NM_001139509.1	363-462	
Nrros	NM_146069.4	3286-3385	
Pdcd1	NM_008798.1	1135-1234	
Plk1	NM_011121.3	1481-1580	
Plk4	NM_011495.2	1661-1760	
Polr2a	NM_009089.2	2221-2320	Housekeeping
Ppic	NM_008908.4	136-235	
Prkca	NM_011101.3	6966-7065	
Pten	NM_008960.2	5161-5260	
Ptpn14	NM_008976.2	967-1066	
Ptpn5	NM_001163565.1	2771-2870	
Pttg1	NM_001131054.1	289-388	
Rorc	NM_011281.2	649-748	
Rpl19	NM_009078.1	21-120	Housekeeping
Slamf1	NM_013730.4	923-1022	
Slamf6	NM_030710.2	806-905	
Slamf8	NM_029084.3	1367-1466	
Slc15a2	NM_021301.2	2216-2315	
Slc16a10	NM_001114332.1	649-748	
Slc16a3	NM_001038653.1	1106-1205	
Slc1a4	NM_018861.3	2136-2235	

HUGO Gene	Accession	Position	Ect
Slc1a5	NM_009201.2	1705-1804	
Slc2a6	NM_172659.2	1411-1510	
Slc37a2	NM_020258.4	1287-1386	
Slc43a1	NM_001083809.1	577-676	
Slc43a3	NM_021398.3	1297-1396	
Slc6a16	NM_001324535.1	2321-2420	
Slc7a10	NM_017394.4	1055-1154	
Smad1	NM_008539.3	241-340	
Smad4	NM_008540.2	2886-2985	
Smad7	NM_001042660.1	3558-3657	
Smad9	NM_019483.4	2574-2673	
Sostdc1	NM_025312.3	722-821	
Sqle	NM_009270.3	1931-2030	
Stat1	NM_009283.3	1591-1690	
Stat3	NM_213659.2	1361-1460	
Stat5a	NM_011488.2	1546-1645	
Stat5b	NM_011489.3	4856-4955	
Tbx21	NM_019507.2	545-644	
Tlr7	NM_133211.3	3211-3310	
Tlr9	NM_031178.2	1802-1901	
Tnf	NM_013693.2	515-614	

Supplementary Table 3. Antibodies information

Target Antigen	Conjugatin	Clone	vendor	Catalog #	Dilution
Bcl2	PE/Cy7	BCL/10C4	BioLegend	633511	1:100
Bcl-6	Alexa Fluor 647	K112-91	BD Biosciences	561525	1:100
CD4	Alexa Fluor 700	GK1.5	eBiosciences	56-0041	1:100
	APC	RM4-5	BioLegend	100516	1:100
	eFlour 450	GK1.5	eBiosciences	48-0041	1:100
	FITC	RM4-5	BD Biosciences	553047	1:100
CD8a	PerCP/Cy5.5	53-6.7	BioLegend	100734	1:200
CD16/CD32	Purified	2.4G2	BD Biosciences	553140	1:500
CD45R/B220	PE-CF594	RA3-6B2	BD Biosciences	562290	1:100
CD44	PE	IM7	Biolegend	103008	1:100
	PE/Cy7	IM7	Biolegend	103030	1:100
	V500	IM7	BD Biosciences	560780	1:100
CD62L	APC	MEL-14	BD Biosciences	553152	1:100
CD69	PE/Cy7	H1.2F3	BioLegend	104512	1:100
CD95	Biotin	Jo2	BD Biosciences	554256	1:100
CD98	PE	RL388	eBiosciences	12-0981	1:100
CD127 (IL-7R)	BV421	A7R34	BioLegend	135027	1:50
CD138	APC	281-2	Biolegend	142506	1:100
CD154 (CD40L)	APC	MR1	BioLegend	106510	1:100
CD162	Alexa Fluor 647	2PH1	BD Biosciences	562806	1:100
	BV510	2PH1	BD Biosciences	563448	1:100
CD278 (ICOS)	PE	7E.17G9	BD Biosciences	552146	1:100
CD279 (PD-1)	eFlour 450	RMP1-30	eBioscience	48-9981	1:100
CXCR5	Purified	2G8	BD Biosciences	551961	1:100
FOXP3	PE	FJK-16s	eBioscience	12-5773	1:100
GL7	eFluor 450	GL-7	eBioscience	48-5902	1:100
Hif-1α	Alexa Fluor700	241812	R&D Systems	IC1935N	1:20
IFN-y	Pacific Blue	XMG1.2	BioLegend	505818	1:100
IL-10	PE/Cy7	JES5-16E3	BioLegend	505026	1:100
IL-17A	PE	TC11-18h10.1	Biolegend	506-904	1:100
KLRG1	PE/Cy7	2F1	Biolegend	138415	1:1000
Ki-67	PE/Cy7	SolA15	eBioscience	25-2698	1:1000
pStat 3 (pY705)	PE	LUVNKI.A	eBioscience	12-9033	1:25
pS6	Alexa Fluor 647	D57.2.2E	Cell Signaling	4851S	1:100
IgD[b]	FITC	217-170	BD Biosciences	553510	1:100
Streptavidin	PE/Cy7		BD Biosciences	5126578	1:100
	PerCP		BD Biosciences	554064	1:100

Supplementary Table 4. Primer sequences for qPCR

Gene Name	Forward (5′ – 3′)	Reverse (5′ – 3′)	Product (bp)
Slc1a4 (*Asct1*)	AGCAGCCATCTTCCAGTGTG	CCGTGGCAGTCACTAGAATGG	101
Slc2a6 (*Glut6*)	CTCTACGCCCTTGGTCTGC	ATCATGATGAGAACCGGCCC	74
Slc7a10 (*Asc-1*)	CCAAGTCTGGTGGGGACTAC	CAGAGCAGTAGGAATCCAGCC	70
Slc16a10 (*Mct10*)	CGTGAGTGTCTTCACGGACA	GAGGCTCGATGGAGCTTACA	110
Slc37a2	CAGGGCTCATCAGCGACTAC	GGAACATCATGGGAGCAGCC	81
Slc16a3 (*Mct4*)	TCACGGGTTTCTCCTACGC	GCCAAAGCGGTTCACACAC	167
Hif1a	AGCTTCTGTTATGAGGCTCACC	TGACTTGATGTTCATCGTCCTC	391
Ppia	GCTGTTTGCAGACAAAGTTCCA	CGTGTAAAGTCACCACCCTGG	138
Hmbs	AGATGGGCAACTGTACCTGAC	GGATGGTGGCCTGCATAGTC	83
